# The African Wastewater Resistome: Identifying Knowledge Gaps to Inform Future Research Directions

**DOI:** 10.3390/antibiotics12050805

**Published:** 2023-04-24

**Authors:** Akebe Luther King Abia, Themba Baloyi, Afsatou N. Traore, Natasha Potgieter

**Affiliations:** 1 One Health Research Group, Biochemistry & Microbiology Department, University of Venda, Private Bag X5050, Thohoyandou 0950, South Africa; thembabaloyi17@gmail.com (T.B.); afsatou.traore@univen.ac.za (A.N.T.); 2 Environmental Research Foundation, Westville 3630, South Africa

**Keywords:** low- and middle-income countries, environmental health, public health, wastewater monitoring, antimicrobial resistance, antibiotic-resistant bacteria, antibiotic resistance genes, wastewater-based epidemiology

## Abstract

Antimicrobial resistance (AMR) is a growing global public health threat. Furthermore, wastewater is increasingly recognized as a significant environmental reservoir for AMR. Wastewater is a complex mixture of organic and inorganic compounds, including antibiotics and other antimicrobial agents, discharged from hospitals, pharmaceutical industries, and households. Therefore, wastewater treatment plants (WWTPs) are critical components of urban infrastructure that play a vital role in protecting public health and the environment. However, they can also be a source of AMR. WWTPs serve as a point of convergence for antibiotics and resistant bacteria from various sources, creating an environment that favours the selection and spread of AMR. The effluent from WWTPs can also contaminate surface freshwater and groundwater resources, which can subsequently spread resistant bacteria to the wider environment. In Africa, the prevalence of AMR in wastewater is of particular concern due to the inadequate sanitation and wastewater treatment facilities, coupled with the overuse and misuse of antibiotics in healthcare and agriculture. Therefore, the present review evaluated studies that reported on wastewater in Africa between 2012 and 2022 to identify knowledge gaps and propose future perspectives, informing the use of wastewater-based epidemiology as a proxy for determining the resistome circulating within the continent. The study found that although wastewater resistome studies have increased over time in Africa, this is not the case in every country, with most studies conducted in South Africa. Furthermore, the study identified, among others, methodology and reporting gaps, driven by a lack of skills. Finally, the review suggests solutions including standardisation of protocols in wastewater resistome works and an urgent need to build genomic skills within the continent to handle the big data generated from these studies.

## 1. Introduction

Antimicrobial resistance (AMR) has been recognised by countries and organisations worldwide as one of the biggest threats to public health in recent times [1,2,3]. It is estimated that without appropriate preventive or remedial measures, the world may experience approximately 10 million losses of lives and over USD 100 trillion annually in the global economy by 2050 [4].

Although micro-organisms possess intrinsic resistance to naturally occurring stressors, the indiscriminate use of pharmaceuticals has been recognised as the most significant contributor to acquired resistance in these organisms, thus escalating the threat to human health [5,6]. For example, the massive and increasing demand for animal protein has engendered an unparalleled use of antibiotics in food animal production, which in 2017 was estimated at 93,309 tons per year globally, with an expected 11.5% increase by 2030 [7]. Furthermore, in humans, misdiagnosis of infections results in the inappropriate prescription of many antibiotics [8]. Therefore, to curb this ill, the World Health Organization (WHO) has identified critical factors driving AMR, including the abusive use of these pharmaceuticals, nonavailability of clean water, sanitation and hygiene (WASH) for human and animal use, inadequate measures to control and prevent infections and diseases in health and animal production settings, inaccessibility to good, and cost-effective medications, vaccines and test procedures, unawareness and lack of knowledge regarding the problem, and nonenforcement of legislation [9].

However, a considerable proportion of the antibiotics consumed by humans and animals are mostly excreted in partially or completely unmetabolised forms, usually containing active ingredients [10,11]. This results in the inevitable discharge of these pharmaceutically active compounds into the environment, especially water bodies, with the major consequence being the potential selection for the survival of resistant micro-organisms. With this, wastewater treatment plants (WWTPs) have been recognised as being among the hotspots for the discharge of antibiotics, their residues and antibiotic-resistant bacteria into the environment [12,13,14,15,16,17]. 

Despite the perceived role of these WWTPs on the spread of AMR, studies evaluating their impact are limited, especially in low- and middle-income countries (LMICs) such as South Africa, where such facilities are usually nonfunctional or function sub-optimally. Furthermore, where such studies are available, the link between environmental and clinical isolates is not apparent, probably because of the basic analyses performed that usually have low discriminating powers to establish such associations. Moreover, the lack of proper reporting of findings influences the acquisition of such data in the public domain. Thus, the present review evaluated the existing literature on AMR in Africa between 2012 and 2022, emphasising South Africa as a case study, to identify gaps that need to be filled to inform future preventive and mitigation measures towards AMR.

## 2. Overview of African Studies between 2012 and 2022

In Africa, the prevalence of AMR in wastewater is of particular concern due to the inadequate sanitation and wastewater treatment facilities, coupled with the overuse and misuse of antibiotics in healthcare and agriculture. African countries, especially in the sub-Saharan region, have the highest disease burdens in the world, with infectious diseases accounting for over 227 million healthy life years and over USD 800 billion yearly productivity loss globally [18]. The ripple effect of this health situation has been identified as the primary factor driving the excessive rate of antimicrobial prescriptions within the continent [19]. For example, consumption of antibiotics in the WHO Watch list increased by 165% in LMIC (including African countries) compared to approximately 28% in their high-income counterparts between 2000 and 2015 [19].

This high antibiotic use implies that wastewater in these countries would be rich in antibiotic residues, antibiotic-resistant bacteria (ARB) and their associated antibiotic-resistance genes (ARGs). For example, a study in Ghana investigated resistance genes, mobile genetic elements (MGEs), from drainage and canalizations before and after three hospitals and an urban waste treatment plant [20]. The main idea was to establish the relationship between the hospital and the wastewater resistome. The authors used a combination of culture-dependent and independent methods, including high-throughput whole-genome sequencing on two sequencing platforms, Nanopore (long reads) and Illumina (short reads). The authors recorded higher resistance rates to carbapenems in the canalization after the hospitals, indicating that the hospital wastewater contributed significantly to the dissemination of resistant bacteria in the environment. Furthermore, the study identified several carbapenemase/β-lactamase genes, including novel variants, such as *bla*_DIM-1_, *bla*_VIM-71_, *bla*_CARB-53_, and *bla*_CMY-172_, with some of these genes associated with MGEs, meaning that these could easily be transferred within and between bacterial communities.

In Nigeria, Akpan et al. [21] isolated Gram-negative bacteria from an abattoir’s wastewater and tested them for antibiotic resistance against five antibiotics, to determine the impact of the abattoir on the environmental resistome. The organisms isolated included *Salmonella* spp., *E. coli*, *Klebsiella* spp., *Shigella* spp., *Pseudomonas* spp. and *Enterobacter* spp. The authors observed that a significant proportion of the isolates (~67%) were resistant to all antibiotics tested, with a 77% multidrug resistance recorded across the samples. However, no extended-spectrum β-lactamase (ESBL)-producing traits were observed in any of the isolates. This study demonstrated that abattoirs contributed considerably to AMR in the aquatic environment.

Tesfaye et al. [22] investigated antimicrobial resistance in *Enterobacteriaceae* in wastewater collected from health settings, an abattoir, and a WWTP, including downstream of a river in Addis Ababa, Ethiopia. The authors obtained 54 isolates, including *E. coli*, *Salmonella* spp., *Klebsiella pneumoniae*, *Enterobacter aerogenes*, *Citrobacter* spp., *Klebsiella oxytoca* and *Enterobacter cloacae*. Antibiotic susceptibility testing revealed that all the isolates were multidrug resistant, while 2 isolates were resistant to all the 12 antibiotics tested. ESBL production was also recorded in 27.3% of the resistant isolates. Furthermore, the hospital wastewater had a higher percentage of resistance than all the other sites, again identifying hospital wastewater as a hotspot for AMR dissemination.

A major shortcoming in all the studies reviewed is that most of them focused on a one-off sampling, usually resulting in a very limited number of isolates or samples. Such small sample sizes would make it challenging to draw strong conclusions and would require further investigations. Furthermore, many studies used either culture or sequencing and only a few used both methods. Using only the culture methods could underestimate the microbial load due to viable but non-culturable isolates, hence reducing the actual resistome reported. On the other hand, using only genomic approaches could overestimate the risk associated with AMR in wastewater. Nevertheless, the presence of any resistance genes and MGEs would signify the possible transmission to other related or even unrelated species. A summary of some studies on wastewater resistome in Africa is provided in Table 1.

**Figure 1:**
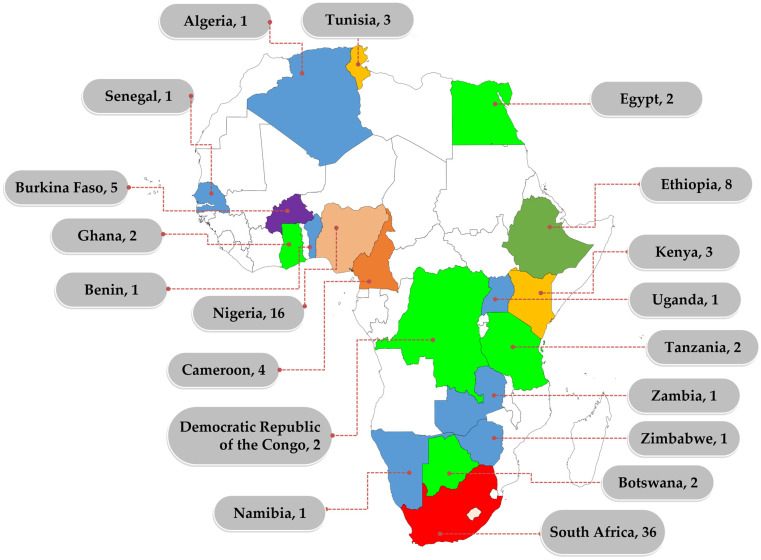
Distribution of African studies on AMR in wastewater between 2012 and 2022. Numbers represent the number of studies identified within the reviewed period. Only counties that reported at least one study in the review period are labelled.

Despite the recognised role of WWTPs in AMR, studies on AMR in wastewater are not evenly distributed within the continent, with most of the studies reported in South Africa (Figure 1).

**Figure 2:**
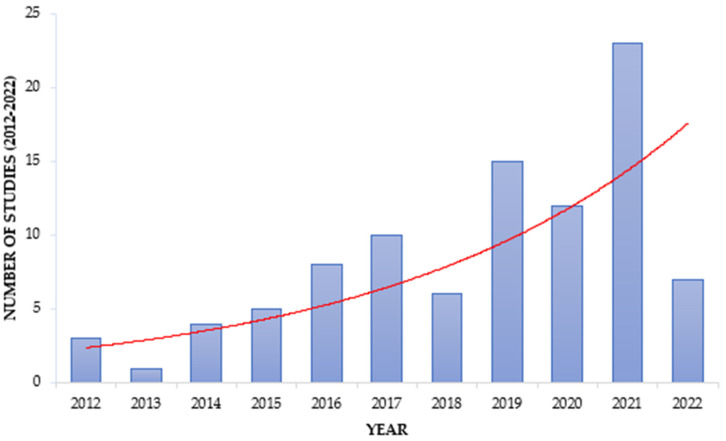
Trend in ARM studies focusing on wastewater. The red line shows the increasing trend within the reviewed period.

However, it is evident that wastewater as a reservoir and source of AMR is gaining attention in Africa, as seen by the increasing trend of studies focusing on wastewater (Figure 2).

## 3. Case Study: South Africa

### 3.1. The South African Wastewater Resistome

A 2015 survey assessed antimicrobial use in inpatients in various hospitals globally and reported that over 50% of African patients received antibiotics [19]. However, a later study revealed a 55% inappropriate use of antimicrobials in some South African primary healthcare facilities [43]. Furthermore, South Africa is among the highest consumers of antimicrobials used in food animals. For example, the country consumed over 870 tons of antimicrobials in food-producing animals, and this quantity is estimated to increase to over 1100 tons by 2030, driven by increased demand for animal protein [19]. These use patterns could be responsible for the AMR rates observed within the country and could ultimately result in a significant discharge of chemically active pharmaceutical residues, ARB and ARGs into the environment through poorly treated or untreated WWTP effluents.

The distribution of WWTPs in South Africa is, Eastern Cape: 123, Free State: 96, Gauteng: 60, KwaZulu-Natal: 147, Limpopo: 64, Mpumalanga: 76, Northern Cape: 78, North-West: 48, and Western Cape: 158 [44]. According to the South African Green Drop evaluation, a WWTP should obtain an overall ≥ 90% Green Drop score to be considered in an excellent functional state [44]. However, according to the 2022 report, the country’s WWTPs have experienced a massive decrease in functional capacity, with the number of WWTPs failing to meet these criteria, significantly increasing from those reported in the preceding report. Thus, monitoring WWTPs would provide an excellent way of determining the AMR burden within the country, and this has attracted interest from the South African scientific community in recent years.

### 3.2. Distribution of Studies by Province

**Figure 3:**
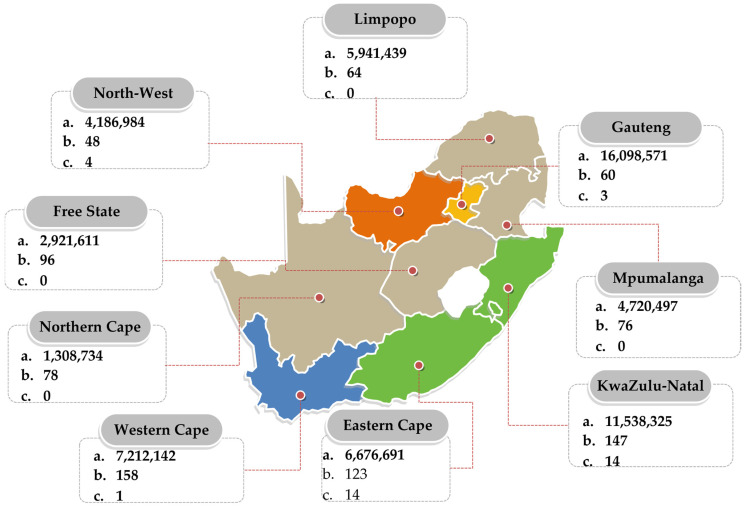
Distribution of South African studies on the wastewater resistome between 2012 and 2022. a = population (https://www.statssa.gov.za/publications/P0302/P03022022.pdf (accessed on 19 April 2023)); b = number of WWTPs in province [44]; c = number of studies.

Several studies have assessed AMR in South African wastewaters. However, a review of the literature between 2012 and 2022 revealed an uneven distribution of the studies within the country’s nine regions, with KwaZulu-Natal and the Eastern Cape accounting for the bulk of the studies identified within the study period (Figure 3).

**Figure 4:**
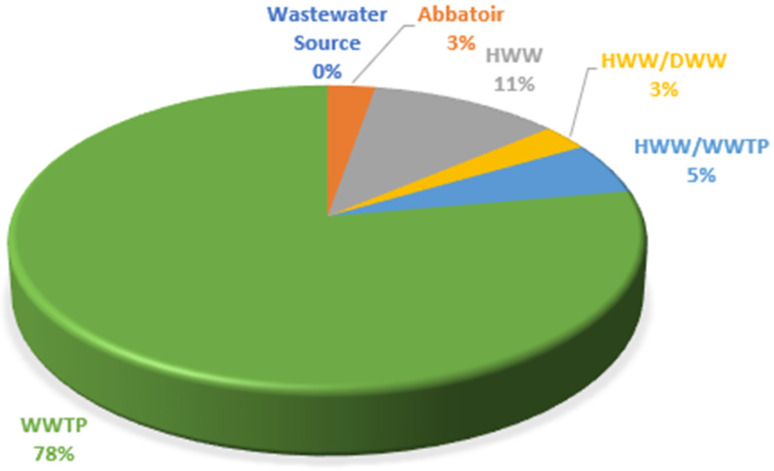
Various wastewater sources evaluated for AMR in South Africa between 2012 and 2022.

Although 36 studies were identified on AMR in wastewater within the study period, not all of them focused on WWTPs (Figure 4). While most of the studies were on WWTPs, other sources of wastewater evaluated included hospital wastewater (HWW), abattoirs and domestic wastewater (DWW).

### 3.3. Micro-organisms Targeted

**Figure 5:**
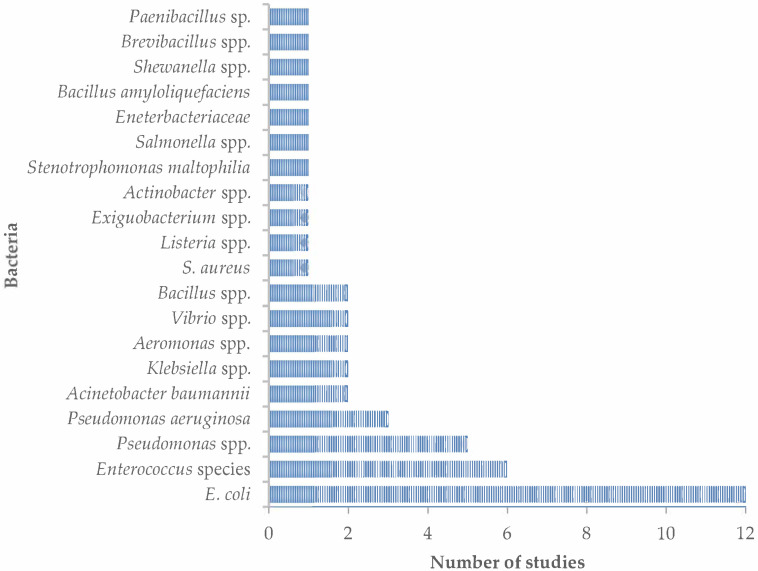
Main micro-organisms identified in South African wastewater (2012–2022).

Microbial species in wastewater are diverse, and attempting to identify them all would not be practical, timewise, resource-wise or technically. Thus, using indicator organisms has been the gold standard for determining the microbial quality of microbially contaminated waters [45,46,47,48,49]. Apart from being a good faecal indicator, *Escherichia coli* has been identified as a good indicator of AMR in the environment, including wastewater [50]. Thus, in the current report, *E. coli* was the most identified organism in all the studies evaluated (Figure 5). However, the culture methods and media used for the identification of *E. coli* and other organisms differed considerably between studies (Table 2).

### 3.4. AMR Determination Methods

The methods used to determine AMR in wastewater samples depend on the aim of the study. Determination of phenotypic resistance is performed using the disk diffusion, agar dilution or broth dilution method [80]. Although disk diffusion is commonly used, automated systems using mainly the broth dilution method have been developed. An example is the VITEK system [81,82].

On the other hand, genotypic resistance is achieved through polymerase chain reaction (PCR) using specific primers to target specific genes [83]. However, this method could be time-consuming and labour-intensive when dealing with many organisms and may require further sequencing of amplified genes to further differentiate them, like with the *tet* genes conferring resistance to tetracycline [60]. Furthermore, recent advances in molecular techniques have allowed the detection of resistance genes in whole populations directly from environmental samples without the need for culture [84].

Finally, whole-genome sequencing (WGS) has been used in cases where high-resolution characterisation of specific isolates is required, as this approach can lead to the identification of novel genes and mutations related to AMR [85].

In the studies reviewed in the current report, the most used method was disk diffusion as most studies focused on phenotypic resistance. Furthermore, the disk diffusion is cost-effective, and flexible, allowing visual growth observation, correct inoculum, mixed (contaminated) cultures and other irregularities [86]. Although the broth dilution method has the added advantage of providing the minimum bactericidal concentration (MBC), the minimum concentration of an antimicrobial that eliminates 99.9% of bacteria [87], this method is more valuable in clinical settings where treatment is required. This could influence its reduced use in the studies evaluated here, as they focused on environmental samples. Where genotypic resistance was investigated, this was mostly achieved through PCR (conventional and real-time). Only a few studies used metagenomics or WGS. There is no doubt that WGS provides an unprecedented level of detail regarding AMR, something that cannot be achieved with culture and other molecular techniques [88]. However, the cost of sequencing and the need for highly skilled bioinformaticians are major impediments to its routine use within the African continent. The VITEK automated system was only used for isolate identification and not for the determination of AMR. Although this system is highly automated and time-efficient, allowing the simultaneous analysis of hundreds of samples [87], the cost of instrumentation could be challenging for most researchers in Africa due to a lack of sufficient research funds. A summary of South African studies that focused specifically on WWTPs between 2012 and 2022 is provided in Table 3.

### 3.5. Water Research Funding

One of the driving factors in research is the availability of funds. For example, the Water Research Commission (WRC) funds most water-related projects in South Africa. This section identifies past WRC projects, and their main aims, to identify similar studies that have been reported on AMR in WWTPs (Table 4). Based on their database, of all these studies, only one focused on antimicrobial resistance in WWTPs (https://search.wrc.org.za/#!/ (accessed on 3 February 2023)). This archive revealed that only a single project was specifically funded relating to the wastewater resistome.

## 4. Identifying Knowledge Gaps

### 4.1. Spatial (Geographical) Gaps

Studies on the WWTP resistome in South Africa have been dominated by two provinces—KwaZulu-Natal and Eastern Cape. Very few studies have been conducted in provinces such as the North-West and Gauteng, while others such as Mpumalanga and Limpopo did not perform such studies within the reviewed period. This provides an incomplete picture of the country’s WWTP resistome. This gap could be due to the non-functioning of most WWTPs in these locations, especially in rural settings.

### 4.2. Methodological Gaps and Associated Challenges

The sampling frequency is not standardised; lower samples may exclude seasonal variation. Infectious diseases requiring antimicrobial treatment, such as diarrhoea usually follow a seasonal pattern [90]. This means that antibiotic consumption would vary based on these seasons. This could therefore affect the type and frequency of resistance observed in wastewater. One-off samplings recorded by Gumede et al. [60] would paint an incomplete picture of the wastewater resistome.

On the other hand, Molale-Tom and Bezuidenhout [70] sampled in a single month (May), while Mbanga et al. [68] sampled for seven months, cutting across different seasons, although both studies focused on *Enterococcus* spp. Furthermore, WWTPs experience periods of peak and low flow [91]. The sampling time could therefore affect the abundance and frequency of AMR, which would be missed with limited sampling. However, none of the studies reviewed indicated the sampling times.

The number and the type of antibiotics tested vary per study, even when the same organisms were tested. For example, Gumede et al. [60], Adegoke et al. [61], Pillay and Olaniran [62], Adefisoye and Okoh [64], and Nzima et al. [65] tested 23, 8, 13, 17 and 9 antibiotics, respectively, although they were all working on *E. coli.* Furthermore, Adegoke et al. [61] tested for colistin which was not tested by the other studies, while Pillay and Olaniran [62] included norfloxacin and fosfomycin in their panel.

These two factors would pose a significant challenge when comparing different studies.

The studies reviewed indicated that the most used detection method was disk diffusion and, in some cases, combined with PCR. However, this creates a knowledge gap regarding the various genes implicated in the observed phenotypic resistance. Although it has been shown that discrepancies exist between phenotypic and genotypic resistance, some organisms may be phenotypically susceptible to the tested antibiotics yet possess genes that could be expressed under appropriate environmental stress, as observed in WWTP settings.

Moreover, culture-based approaches would introduce selection bias, as only a subset of isolates is usually selected for downstream analysis. This would also be the case with WGS, where a selected number of isolates would be subjected to sequencing. On the other hand, metagenomic approaches would identify genes in a total population, regardless of the micro-organisms. Despite the advantages of genomic methods for AMR monitoring, these methods were only used in very few studies during the review period.

This methodological gap is probably fuelled by two main factors: the cost of performing advanced genomic studies and the lack of technical skills, including bioinformatic skills for analysing genomic data.

### 4.3. Micro-organisms Gap

Gram-positive and Gram-negative bacteria differ in the structure of their outer membranes, a characteristic that affects their response to antibiotics. Thus, because of an extra outer layer, Gram-negative bacteria have been reported to be more antibiotic-resistant than their Gram-positive counterparts [92,93]. However, most of the evaluated studies focused on *E. coli* (Gram-negative), while a few assessed *Enterococcus* spp. (Gram-positive).

Despite the greater medical importance of Gram-negatives, Gram-positive bacteria could serve as important reservoirs of ARGs within WWTPs. This reliance on *E. coli* alone is also due to the simplicity of its isolation and characterisation, which make it a suitable organism for monitoring AMR. However, determining the WWTP resistome using *E. coli* alone could lead to gross underestimation of AMR in these milieus.

### 4.4. Reporting Gap

Research findings should be made available for consumption by the general public and relevant stakeholders as this would foster the implementation of such findings for the benefit of humanity and its environment [94,95]. However, while the studies reviewed here were journal articles published in scholarly outlets, such information does not usually get to the grassroots people, who are more impacted by the problems investigated. Furthermore, even with the scientific publications, the analysis gaps identified earlier significantly affect the overall information available on AMR in WWTPs due to the non-standard nature of the studies. For example, repositories containing the various resistances identified in the studies are unavailable within the country.

## 5. Proposed Future Perspective

It is evident that wastewater-based monitoring of AMR is gaining significant ground globally, including in South Africa. However, this could still be challenging in many African countries as most LMICs lack structured sewer systems. However, in places such as South Africa where such facilities are available:

(i). There is a need to standardise protocols for assessing the WWTP resistome. This should consider the sampling regime, the sampling frequency, the organisms targeted, which antibiotics need to be tested and which methods should be used.
(ii). There is a need to build capacity in sequencing technologies and bioinformatics, given the recent drift of the science to big data analysis.
(iii). Funding must be made available to researchers as sequencing technologies are not yet widespread in the country, and the cost of using these facilities is still considerably high.
(iv). Reporting of works on AMR in WWTPs needs to be improved, and there is a need to create a repository that would serve as a referral point for future studies.

## Figures and Tables

**Table 1:** Summary of some studies on AMR in wastewater in Africa between 2012 and 2022.

Country	^&^ Wastewater Type/Source	Duration of Study	Sample Size	Targeted Resistance	Phenotypic (P)/Genotypic (G) Resistance	Method	Reference
* South Africa	WWTP	Two campaigns—actual duration not mentioned	^#^ Not indicated	Cefotaxime-resistance	P	Culture	[23]
Algeria	WWTP	3 days in 2 months	Not indicated	ESBLs and associated quinolone resistance	P, G	Culture; PCR	[24]
Botswana	WWTP	^$^ One-off sampling	one	Overall resistome	G	Shotgun metagenomics	[25]
Botswana	WWTP	Monthly for 1 year	72	General resistance—9 antibiotics tested	P	Culture	[26]
Burkina Faso	Urban channel	6 months	101	ESBLs	P	Culture	[27]
Burkina Faso	WWTP	Monthly for 5 months	15	General resistance—19 antibiotics	P	Culture	[28]
Cameroon	Open-air canals	One-off	6 (composite) samples	Overall resistome	G	Shotgun metagenomics	[29]
Ethiopia	Hospital wastewater	3 months	27	General resistance—13 antibiotics	P	Culture	[30]
Ethiopia	Hospital wastewater	4 months	40 (composite samples)	General resistance—13 antibiotics	P	Culture	[31]
Ghana	WWTP	Monthly—6 months	30	General resistance	P	Culture	[32]
Kenya	University WWTP	4 months	Not mentioned	Overall resistome	P, G	Culture; whole-genome sequencing	[33]
Kenya	Septic tank	2 months	Not mentioned	General resistance	P	Culture	[34]
Kenya	WWTP	6 months (covering the dry and rainy seasons)	24	General resistance	P	Culture	[35]
Nigeria	Hospital WWTP	Weekly for 4 months	Not mentioned	ESBLs	P, G	Culture; PCR	Adekanmbi
Senegal	Slaughterhouse wastewater and WWTP	Not mentioned	Not mentioned	General resistance—16 antibiotics	P	Culture	[36]
South Africa	WWTP	7 months (Every two weeks)	81	Overall resistome	P, G	Culture; whole-genome sequencing	[37]
Tanzania	WWTP	2013/2014 (Not specific)	52	General resistance—14 antibiotics	P	Microdilution	[38]
Tunisia	WWTP	Not mentioned	Not mentioned	*intI1*, ARGs *bla*_CTX-M_, *bla*_TEM_, *qnr*A, *qnr*S, *sul* I, *erm*B	G	PCR	[39]
Uganda	Multiple sources	Not mentioned	Not mentioned	General resistance—15 antibiotics	P	Culture	[40]
Zambia	Wastewater ponds	Not mentioned	5 samples	General resistance—8 antibiotics	P	Culture	[41]
Zimbabwe	Abattoir wastewater	3 months	600 samples	General resistance—16 antibiotics	P	Culture	[42]

* Part of a multinational (22 countries) study in Europe, Asia, Africa, Australia, and North America. ^#^ A total of 472 samples were collected from all the countries. ^$^ Analysed once and used to irrigate soil. Focus was not on the monitoring of the wastewater resistome, but the impact of the wastewater in the soil resistome. ^&^ Includes influent or effluent or both.

**Table 2:** Media and incubation conditions used for the identification of different micro-organisms in waterwater AMR studies in South Africa between 2012 and 2022.

Organism	Media	Incubation Temperature (°C)	Duration (Hours)	Reference
*Brevibacillus* spp.; *Paenibacillus* spp.	R2A media	Not mentioned (NM)	NM	[51]
*Acinetobacter baumannii*	Leeds Acinetobacter Medium	37	24	[52]
*Acinetobacter baumannii*; *Acinetobacter* spp.	CHROMagar Acinetobacter	37	18–24	[53,54]
*Aeromonas*, *Exiguobacterium*	Nutrient agar, Blood agar	NM	NM	[55]
*Aeromonas* spp.	Glutamate Starch Phenol-red (GSP) agar plates	37	24	[56]
*Aeromonas* spp.	Rimler-Shotts agar	37	20	[57]
*Aeromonas* spp.	Aeromonas spp. Isolation agar	37	24	[58]
*Bacillus amyloliquefaciens*	nutrient agar	37	18–24	[59]
*Bacillus* spp.	Nutrient agar, Blood agar	NM	NM	[55]
*Bacillus* spp.	R2A media	NM	NM	[51]
*E. coli*	Eosin methylene blue agar	37	24	[60]
*E. coli*	Membrane Fecal Coliform (mFC) agar supplemented with 4 mg/L or 8 mg/L cefotaxime antibiotic	37	24	[61]
*E. coli*	Chromocult Coliform Agar (Merck)	37	24	[62]
*E. coli*	*E. coli*-Coliforms Chromogenic medium	37	24	[63,64]
*E. coli*	CHROMagar ECC	37	24	[65]
*E. coli*	*E. coli*-coliform selective agar	37	24	[66]
*E. coli*	Chromogenic agar *	37	24	[67]
*E. coli*	Colilert-18^TM^	37	24	[68]
*Enterobacteriaceae*	Violet Red Bile Glucose (VRBG) agar	37	18	[69]
*Enterococcus* spp.	R2A media	NM	NM	[51]
*Enterococcus* spp.	KF-Streptococcus agar containing 1 mL of 2,3,5-Triphenyltetrazolium chloride	37	48	[70]
Enterococcus spp.	chromogenic 51,759 HiCrome™ Rapid Enterococci Agar media	37	24–48	[71]
*Enterococcus* spp.	Tryptic Soy Broth	37	18	[67]
*Enterococcus* spp.	Bile Aesculin Azide Agar	37	24	[67]
*Enterococcus* spp.	CHROMagar™ VRE, BBL™ Enterococcosel™ Broth	37 ± 2 °C	18 to 24	[72]
*Enterococcus* spp.	Enterolert^TM^	41	24–48	[68]
*Klebsiella* spp.	Nutrient agar, Blood agar	NM	NM	[55]
*Klebsiella* spp.	HiCrome Klebsiella selective agar	35	24	[73]
Listeria spp.	Listeria Chromogenic agar	35	24–48	[57]
*Pseudomonas aeruginosa*	Mineral salt medium	30	18–24	[59]
*Pseudomonas aeruginosa*	CHROMagarTM Pseudomonas	37	24–48	[74]
*Pseudomonas* spp.	Nutrient agar, Blood agar	NM	NM	[55]
*Pseudomonas* spp.	R2A media	NM	NM	[51]
*Pseudomonas* spp.	Pseudomonas Isolation Agar	35	24–48	[75]
*Pseudomonas* spp.	Cetrimide agar	37	24	[58]
*Pseudomonas* spp.	Glutamate Starch Phenol-red (GSP) agar	37	24	[56]
*Salmonella* spp.	Salmonella-Shigella (SS) agar	37	24–48	[76]
*Shewanella* spp.	Nutrient agar, Blood agar	NM	NM	[55]
*Staphylococcus aureus*	Mannitol Salt Agar supplemented with cefoxitin.	Not mentioned (NM)	NM	[77]
*Stenotrophomonas maltophilia*	Stenotrophomonas selective agar base with Vancomycin Imipenem Amphotericin B (VIA) supplement	37	18 to 24	[54]
*Vibrio* spp.	thiosulfate-citrate–bile salt-sucrose (TCBS) agar	37	24	[63,78,79]

* Specific media was not mentioned.

**Table 3:** Summary of AMR studies on WWTPs in South Africa (2012–2022).

Organism(s)	Antibiotics Tested (*n* = Number Tested)	Phenotypic Resistance	Genotypic Resistance	Method	Reference
*E. coli*	*n* = 23: Amoxicillin/clavulanic acid, amoxicillin, amikacin, ampicillin, cefepime, cephalothin, cefotaxime, cefoxitin, cefixime, nalidixic acid, ceftazidime, cephalexin, cefuroxime, chloramphenicol, ciprofloxacin, gentamicin, imipenem, meropenem, nitrofurantoin, piperacillin, tetracycline, tigecycline, trimethoprim/Sulfamethoxazole.	Amoxicillin/clavulanic acid, amoxicillin, amikacin, ampicillin, cefepime, cephalothin, cefotaxime, cefoxitin, cefixime, ceftazidime, cephalexin, cefuroxime, chloramphenicol, ciprofloxacin, gentamicin, imipenem, meropenem, nitrofurantoin, piperacillin, tetracycline, tigecycline, nalidixic acid, trimethoprim/Sulfamethoxazole.	*TEM*, *SHV*, CTX-M	DD/PCR-Sanger Sequencing	[60]
*E. coli*	*n* = 8: Meropenem, colistin, amoxicillin/clavulanic, ciprofloxacin, nitrofurantoin trimethoprim/sulfamethoxazol, gentamicin, tetracycline.	Colistin, amoxicillin-clavulanic, ciprofloxacin, trimethoprimsulphamethoxazole, gentamicin, tetracycline, nitrofurantoin.	*TEM*, *SHV*, CTX-M, *VIM*, *OXA-*1, *KPC*-2, *NDM*-1	DD/PCR	[61]
*S. aureus*	*n* = 20: Amikacin, Gentamicin, Amoxicillin/clavulanic acid, Ampicillin, Oxacillin, Penicillin, Imipenem, Cefoxitin, Cefozolin, Ciprofloxacin, Norfloxacin, Vancomycin, Clindamycin, Lincomycin, Azithromycin, Erythromycin, Chloramphenicol, Rifampicin, Tetracycline Sulfamethoxazole/trimethoprim.	Amikacin, Gentamicin, Amoxicillin/clavulanic acid, Ampicillin, Oxacillin, Penicillin, Imipenem, Cefoxitin, Cefozolin, Norfloxacin, Vancomycin, Clindamycin, Lincomycin, Azithromycin, Erythromycin, Chloramphenicol, Rifampicin, Sulfamethoxazole/trimethoprim, Tetracycline.	*aac(6′)/aph(2″), bla*Z*, erm*C, *msr*A and *tet*K,	DD/PCR	[77]
*Klebsiella* spp.	*n* = 16: Amoxicillin-clavulanic acid, piperacillin-tazobactam, cefotaxime, ceftazidime, cefalexin, cefoxitin, ertapenem, meropenem, doripenem, imipenem, aztreonam, ciprofloxacin, norfloxacin, moxifloxacin, gentamicin, tobramycin.	Amoxicillin-clavulanic acid, piperacillin-tazobactam, cefotaxime, ceftazidime, cefalexin, cefoxitin, ertapenem, doripenem, aztreonam, ciprofloxacin, norfloxacin, moxifloxacin, gentamicin, tobramycin.		DD	[73]
*Aeromonas* spp.	*n* = 20: Ciprofloxacin, Trimethoprim, Ofloxacin, Chloramphenicol, Penicillins, Clindamycin, Ampicillin-sulbactam, Ampicillin, Gentamicin, Nalidixic acid, Cefotaxime, Nitrofurantoin, Oxacillin, Sulphamethoxazole, Cephalothin, Erythromycin, Tetracycline, Minocycline, vancomycin, Rifamycin.	Ciprofloxacin, Trimethoprim, Chloramphenicol, Penicillins, Clindamycin, Ampicillin-sulbactam, Oxacillin, Ampicillin, Gentamicin, Nalidixic acid, Cefotaxime, Nitrofurantoin, Sulphamethoxazole, Cephalothin, Erythromycin, Tetracycline, Minocycline, vancomycin, Rifamycin.	blaP1class A β-lactamase (*pse1-PSE-1/CARB-2*), *bla*_TEM_, *Tet*C, Class 1 integron, Class 2 integron	DD/PCR	[56]
*Listeria* spp.	*n* = 24: Penicillin, Cephalothin, Gentamicin, Kanamycin, Amikacin, Ertapenem, Meropenem, Cefotaxime, Ceftriaxone, Vancomycin, Clindamycin, Erythromycin, Nitrofurantoin, Ampicillin, Colistin, Nalidixic acid, Mixofloxacin, Fusidic Acid Ciprofloxacin, Trimethoprim, Tetracycline, Streptomycin, Fosfomycin Chloramphenicol.	Penicillin, Cephalothin, Kanamycin, Ertapenem, Cefotaxime, Ceftriaxone, Vancomycin, Clindamycin, Erythromycin, Nitrofurantoin, Ampicillin, Colistin, Nalidixic acid, Mixofloxacin, Trimethoprim, Tetracycline,		DD	[57]
*Aeromonas* spp.		Penicillin, Cephalothin, Kanamycin, Ertapenem, Meropenem, Cefotaxime, Ceftriaxone, Vancomycin, Clindamycin, Erythromycin, Nitrofurantoin, Ampicillin, Colistin, Nalidixic acid, Mixofloxacin, Trimethoprim, Tetracycline, Streptomycin, Chloramphenicol, Fosfomycin, Fusidic Acid.			
*E. coli*	*n* = 13: Ampicillin, amoxicillin, cephalothin, cefazolin, ceftazidime, tetracycline, doxycycline, chloramphenicol, amikacin, gentamicin, nalidixicacid, norfloxacin, fosfomycin.	Ampicillin, amoxicillin, cephalothin, ceftazidime, tetracycline, doxycycline, chloramphenicol, nalidixic acid, norfloxacin, fosfomycin.		DD	[62]
*Klebsiella* *Bacillus* *Pseudomonas* *Aeromonas Exiguobacterium* *Shewanella* spp.	*n* = 6: Vancomycin, kanamycin, trimethoprim, oxytetracycline, amoxicillin and chloramphenicol.	Vancomycin, kanamycin, trimethoprim, oxytetracycline, amoxicillin and chloramphenicol.		BD	[55]
*Enterococcus* spp.	*n* = 1: Vancomycin		*erm*(B) *was*, VREfm, *van*A (*van*A, *van*HA, *van*RA, *van*SA, *van*YA and *van*ZA gene clusters), *van*G (*van*RG), vanN (*van*RN) and *van*L (*van*SL), *van*C (*van*C1XY, *van*SC, *van*RC and *van*XYC), *isa*(A), *et*(M), *aac(6′)-Ii*	WGS	[72]
*Enterobacteriaceae*	*n* = 18: Doxycycline, tetracycline, ampicillin, gentamicin, meropenem amoxicillin/clavulanic acid, amikacin, nitrofurantoin, cefuroxime, cefotaxime, norfloxacin, ciprofloxacin, chloramphenicol, nalidixic acid, colistin sulphate, polymyxin, trimethoprim-sulfamethoxazole, imipenem.	Gentamycin, neomycin, penicillin G, nitrofurantoin, polymyxin B, cefuroxime.	ESBL (*bla*_CTX-M_, *bla*_TEM_, *bla*_SHV_, *bla*_GES_, *bla*_IMP_, *bla*_KPC_, *bla*_VIM_, *bla*_OXA-1-like_,*bla*_PER_, *bla*_OXA-48-like_, and *bla*_VEB_), pAmpC (bla_ACC_, bla_EBC_, *bla*_FOX_,*bla*_CIT_, *bla*_DHA_, and *bla*_MOX_), non-β-lactam (*aad*A, *cat*I,*cat*II, *str*A, *sul*I, *sul*II, *tet*A, *tet*B, *tet*C, *tet*D, *tet*K, and *tet*M)	DD/PCR	[69]
*E. coli*	*n* = 18: Ampicillin, amikacin, imipenem, meropenem, streptomycin, ciprofloxacin, chloramphenicol, nalidixic, tetracycline, trimethoprim, norfloxacin, Sulfamethoxazole, gentamycin, neomycin, penicillin G, nitrofurantoin, polymyxin B, cefuroxime.		*bla*_TEM_, *bla*_SHV_, *bla*_Z_, *bla*_CTX-M_, *aad*A, *str*A, *tet*A, *tet*B, *tet*K and *tet*M,	DD/PCR	[63]
*Vibrio* spp.		Ampicillin, amikacin, imipenem, meropenem, streptomycin, chloramphenicol, ciprofloxacin, nalidixic, tetracycline, trimethoprim, norfloxacin, Sulfamethoxazole, gentamycin, neomycin, penicillin G, nitrofurantoin, polymyxin B, cefuroxime.			
*Enterococcus* spp.	*n* = 14: Chloramphenicol, tetracycline, ampicillin, nitrofurantoin, ciprofloxacin, levofloxacin, imipenem, linezolid, erythromycin, quinupristin-dalfopristin, tigecycline, trimethoprim-sulfamethoxazole, vancomycin, teicoplanin.		*lsa*(A), *msr*(C), *msr*(D), *erm*(B), and *mef*(A), *tet*(S), *tet*(M), and *tet*(L), *aac(60)-aph(200)*, *ant(6)-Ia*, *aph(30)-*III, *aac(60)-Iid*, *aac(60)-Iih*, *dfr*G	DD/WGS	[37]
*E. coli*	*n* = 17: Ampicillin, amikacin, imipenem, meropenem, streptomycin, cefotaxime, chloramphenicol, cephalexin, ciprofloxacin, nalidixic acid, tetracycline, norfloxacin, gentamicin, cefuroxime, polymyxin B, colistin sulfate, and nitrofurantoin.	Ampicillin, amikacin, streptomycin, chloramphenicol, ciprofloxacin, cephalexin, nalidixic acid, tetracycline, norfloxacin, gentamicin, cefuroxime, cefotaxime, polymyxin B, colistin sulfate, and nitrofurantoin.	*str*A, *aad*A, *cat* I, *cat* II, *cml*A1, *amp*C, *bla*_Z_, *bla*_TEM_, *tet*A, *tet*B, *tet*C, *tet*D, *tet*K, *tet*M	DD/PCR	[64]
*Aeromonas* spp.	*n* = 12: Ampicillin, ceftazidime, cefixime, polymyxin B, colistin, ciprofloxacin, levofloxacin, ofloxacin, minocycline, meropenem, imipenem, trimethoprim-sulphamethoxazole.	Ampicillin, ceftazidime, cefixime, polymyxin B, colistin, ciprofloxacin, levofloxacin, minocycline, meropenem, imipenem, trimethoprim-sulphamethoxazole.	*bla*_TEM_, *bla*_AmpC_, *Amp*C/*_bla_*_OXA_, *mcr-*1,	DD/PCR	[58]
*Pseudomonas* spp.		Ampicillin, ceftazidime, cefixime, polymyxin B, colistin, ciprofloxacin, levofloxacin, ofloxacin, minocycline, meropenem, imipenem, trimethoprim-sulphamethoxazole.			
*Enterococci*			*erm*A,*erm*B and *erm*C, *tet*K, *tet*M and *tet*L, *van*A, *van*B and *van*C, *aph(3‘)-IIIa*, *ant(4‘)-Ia*,*aac(6′)-Ie-aph(2”)-Ia*	PCR	[71]
*Vibrio* spp.	*n* = 13: Imipenem, nalidixic acid, erythromycin, gentamicin, Sulfamethoxazole, cefuroxime, penicillin G, chloramphenicol, polymixin B, trimethoprim-sulfamethoxazole, tetracycline, meropenem and trimethoprim.	Nalidixic acid, erythromycin, Sulfamethoxazole, cefuroxime, penicillin G, chloramphenicol, polymixin B, trimethoprim-sulfamethoxazole, tetracycline and trimethoprim.		DD	[78]
*Salmonella* spp.	*n* = 20: Cephalothin, Imipenem, Cefoxitin, Cefuroxime, Piperacillin, Ampicillin, Cefixime, Ceftazidime, Aztreonam, Gentamycin, Amikacin, Streptomycin, Chloramphenicol, Tetracycline, Ciprofloxacin, Norfloxacin, Nalidixic acid, Nitrofurantoin, Sulfamethoxazole Trimethoprim/Sulfamethoxazole.	Imipenem, Piperacillin, Ampicillin, Cefixime, Ceftazidime, Streptomycin, Nalidixic acid, Sulfamethoxazole.		DD	[76]
*Pseudomonas* spp.	*n* = 19: Ampicillin, cefotaxime, cephalothin, cefepime, chloramphenicol, clindamycin, erythromycin, gentamicin, minocycline, nalidixic acid, nitrofurantoin, ofloxacin, oxacillin, penicillin G, rifampin, sulphamethoxazole, tetracycline, vancomycin, ampicillin-sulbactam.	Ampicillin, cefotaxime, cephalothin, cefepime, chloramphenicol, clindamycin, minocycline, nalidixic acid, nitrofurantoin, oxacillin, penicillin G, rifampin, sulphamethoxazole, tetracycline, vancomycin, ampicillin-sulbactam.		DD	[75]
*Enterococcus* spp.	*n* = 11: Ampicillin, amoxicillin, penicillin, neomycin, streptomycin, vancomycin, chloramphenicol, ciprofloxacin, tetracycline, trimethoprim, erythromycin.	Ampicillin, amoxicillin, penicillin, neomycin, streptomycin, vancomycin, chloramphenicol, ciprofloxacin, tetracycline, trimethoprim, erythromycin.		DD	[70]
*E. coli*	*n* = 9: Ampicillin, penicillin, ciprofloxacin, tetracycline, trimethoprim, cefotaxime, ceftazidime, imipenem and meropenem.	Ampicillin, penicillin, ciprofloxacin, tetracycline, trimethoprim, cefotaxime, ceftazidime.	*Alr*, *bla*_TEM_, *bla*_SHV_ and *bla*_CTX-M_	DD/PCR	[65]
*Bacillus, Pseudomonas, Enterococcus, Brevibacillus, Paenibacillus*	*n* = 3 Penicillin G, vancomycin, erythromycin.	Vancomycin Erythromycin Penicillin G		DD	[51]
*E. coli*	*n* = 12: Amoxicillin, Cefuroxime, Gentamicin, Doxycycline, Ciprofloxacin, Ofloxacin, Trimithoprime, Menopenem, Colistin sulphate, Erythromycin, Clindamycin, Sulphamethoxazole.	Amoxicillin, Cefuroxime, Gentamicin, Doxycycline, Ciprofloxacin, Ofloxacin, Trimithoprime, Menopenem, Colistin sulphate, Erythromycin, Clindamycin, Sulphamethoxazole.		DD	[67]
*Pseudomonas* spp.	*n* = 20: Penicillins, clinamycins, ciprofloxacin, rafamycin, trimethoprim, sulphamethoxazole, gentamicin, chloramphenicol, tetracycline, erythromycin, minocycline, vacomycin, cefotaxime, nalidixic acid, nitrofurantoin, cephalothin, ofloxacin, ampicillin, ampicillin-sulbactam, oxacillin.	Penicillins, clinamycins, rafamycin, trimethoprim, sulphamethoxazole, chloramphenicol, tetracycline, minocycline, vacomycin, cefotaxime, nalidixic acid, nitrofurantoin, cephalothin, ampicillin, ampicillin-sulbactam, oxacillin.	*bla*_TEM_, *bla*_OXA_, *bla*_AmpC_, *Tet*C,	DD/PCR	[89]
*Escherichia coli* *Enterococcus* spp.	*n* = 22: Amikacin, ampicillin, azithromycin, amoxicillin-clavulanic acid, cefepime, cefotaxime, cefoxitin, ceftazidime, ceftriaxone, cephalexin, ciprofloxacin, chloramphenicol, gentamicin, imipenem, meropenem, nalidixic acid, piperacillin-tazobactam, tetracycline, tigecycline, trimethoprim-sulfamethoxazole.				[68]
	*n* = 16: Imipenem, Ampicillin, tetracycline, Nitrofurantoin, quinupristin-dalfopristin, tigecycline, Linezolid, ciprofloxacin, trimethoprim-sulfamethoxazole, Levofloxacin, Teicoplanin, vancomycin, Gentamycin, Streptomycin, Erythromycin, chloramphenicol.				

DD = Disk diffusion; BD = Broth dilution; PCR = Polymerase chain reaction; WGS = Whole-genome sequencing 3.5. Water research funding.

**Table 4:** Past WRC-funded projects.

SN	Report Number	Project Title	Year	Aim	WWTP	AST
1	1126/1/05	Enteric pathogens in water sources and stools of residents in the Venda region of the Limpopo Province	2005	Identify and characterise enteric pathogens in water sources and stool samples of residents in the Venda region of the Limpopo Province	No	Yes
2	1967/1/13	Investigations into the existence of unique environmental *Escherichia coli* populations	2013	Identify and characterise *E. coli* from chosen localities and different samples	No	No
3	2138/1/16	An investigation into the presence of free-living amoebae and amoeba-resistant bacteria in drinking water distribution systems of health care institutions in Johannesburg, South Africa	2016	To establish the occurrence of free-living amoebae and amoeba resistant bacteria within the drinking water distribution system in health care facilities in Johannesburg and also highlight the potential human health risk implication thereof	Yes	No
4	2432/1/18	Cholera Monitoring and Response Guidelines	2018	The development of cholera monitoring and response guidelines for inclusion in the water resource monitoring programme.	Yes	Yes
5	2585/1/19	Antibiotic-resistant bacteria and genes in drinking water. Implications for drinking water production and quality monitoring	2019	Identify and characterise microbial parameters in drinking water systems	No	Yes
6	2610/1/18	Microplastics in freshwater water environments	2018	Identify and characterise microplastics in freshwater, drinking water and groundwater	No	No
7	2706/1/21	Measurement of water pollution determining the sources and changes of microbial contamination and impact on food safety from farming to retail level for fresh vegetables	2021	To determine the link between water pollution and crop contamination and to determine sources of microbial product contamination, and assess the impact on food safety from farming to retail for selected fresh vegetable supply chains	No	Yes
8	2733/1/20	Substances of emerging concern in South African aquatic ecosystems	2020	Identify and evaluate different contaminants of emerging concern in different water sources	Yes	No
9	1655/1/10	Identification of Arsenic Resistance Genes in Micro-organisms from Maturing Fly Ash-Acid Mine Drainage Neutralised Solids	2011	To isolate micro-organisms resistant to arsenic from matured AMD-FA neutralized solids, to characterize their arsenic resistance systems and to assess whether these organisms pose a potential ‘threat’ to the sustained use of ‘Neutralization Solids’	No	No
10	KV 360/16	A Scoping Study on the Levels of Antimicrobials and Presence of Antibiotic-Resistant Bacteria in Drinking Water	2016	To provide an overview of the levels of antimicrobials and the presence of antibiotic-resistant bacteria in selected drinking water treatment systems (drinking water production facilities)	No	Yes
11	TT 742/1/17	Emerging contaminants in wastewater treated for direct potable reuse: the human health risk priorities in South Africa	2018	Identify and evaluate different contaminants of emerging concern in different water sources	Yes	No
12		The epidemiology and cost of treating diarrhoea in South Africa		Identify and characterise enteric pathogens in water sources and stool samples of residents in the Venda region of the Limpopo Province	No	Yes

## Data Availability

Not applicable.
